# Performance evaluation of optimal real-time polymerase chain reaction achieved with reduced voltage

**DOI:** 10.1186/s12938-018-0579-0

**Published:** 2018-11-06

**Authors:** Ji-Soo Hwang, Jong-Dae Kim, Yu-Seop Kim, Hye-Jeong Song, Chan-Young Park

**Affiliations:** 1 Dept. of Computer Engineering, Hallym University, Chuncheon, South Korea; 2 Dept. of Convergence Software, Hallym University, Chuncheon, South Korea; 3 Bio-IT Research Center, Hallym University, Chuncheon, South Korea

## Abstract

**Background:**

Polymerase chain reaction (PCR) is used in nucleic acid tests of infectious diseases in point-of-care testing. Previous studies have demonstrated real-time PCR that uses a micro-PCR chip made of packing tape, double-sided tape, and a plastic cover with polycarbonate or polypropylene on a black matte printed circuit board substrate. Despite the success of DNA amplification and fluorescence detection using an early version of the micro-PCR chip, reaching the target temperature was fairly slow and, as a result, the total running time was getting longer. To reduce this runtime, the micro-PCR chip was modified by reducing the heater pattern size of the PCB substrate to one-quarter of the original size or less, while maintaining the ability of the heating pattern to cover the reservoir area of the microfluidic channel. In subsequent experiments, DNA amplification failed several times. During the analysis of the cause of this failure, it was found that the reagent was boiling with the heating range from 25 to 95 °C.

**Methods:**

As a method of DNA amplification verification, images were captured by digital single-lens reflex camera to detect FAM fluorescence using diagonal illumination from a blue LED light source. The images were automatically captured at 72 °C (the extension step in nucleic acid amplification) and the brightness of the captured images was analyzed to con-firm the success of DNA amplification.

**Results:**

Compared to the previous chip with a larger heating pattern size, the current chip appears to generate excess energy as the size of the heating pattern was reduced. To reduce this excess energy, the initial voltage was lowered to 2 V and 2.5 V, which is equivalent to a one-fifth and one-quarter voltage–power reduction in pulse width modulation control, respectively. In both voltage reduction cases, the DNA amplification was successful.

**Conclusions:**

DNA amplification tests may fail due to the excess energy generated by reducing the heater pattern size of the PCB substrate. However, the tests succeeded when the voltage was reduced to 2 V or 2.5 V. The 2.5 V power test was more efficient for reducing the overall running time.

## Background

Various inexpensive, disposable lab-on-a-chip systems (LOCs) are being developed for the purposes of miniaturizing, integrating, and automating point-of-care diagnoses using routine biochemical processes [1–3]. LOCs are used for various purposes in biotechnology, medical treatment and diagnosis, and basic research [4, 5]. The most urgent issues to address with the use of LOCs are ensuring that the fluid of cells and the aqueous solution of biomolecules are processed stably and inexpensively, and maintaining a favorable process for smaller sample sizes [1, 2, 6, 7]. Because of these requirements, microfluidic channels have been actively developed to process small sample amounts; generally, these channels are integrated through the expensive and difficult processes of etching, baking, and bonding the silicon, polymer, and glass materials [1, 2]. The use of tape, which is a thinner and flexible substitute for a microfluidic channel, allows for more efficient and less expensive channel fabrication [2, 8].

The market for LOCs is expanding greatly. Because mass-produced tapes are available at various thicknesses and they are low cost [2], the thermal cycling required for DNA amplification can be effectively conducted by selecting a thin tape to lower the thermal resistance. Furthermore, because the microfluidic channel is created by simply carving out the tape, fabrication is convenient.

In a previous study, a micro-PCR chip fabricated with double-sided tape produced results similar to those of conventional PCRs [9]. It was also confirmed that real-time PCR could be implemented using the micro-PCR chip based on a black PCB, as suggested in previous studies [10, 11]. Although the heating pattern circuit, which acts as a heater, is sufficiently wide to involve the microfluidic channel, the DNA amplification is successful. This is because the resistance of the heating pattern is high, and it takes time to attain the target temperature using heating and cooling processes.

In order to reduce the time needed to reach the target temperature, we performed PCR by reducing the area of the heater pattern to one-quarter or less of the conventional area. We then compared the results to previous experiments that used a large heating pattern. By reducing the size of the heater pattern, we predicted that the decrease in time to reach the target temperature would have no effect on DNA amplification. However, the amplification of DNA in the real-time PCR test failed. While investigating this amplification failure, we repeated the experiment while the PID control algorithm was being adjusted and found that the reagent in the chip was boiling in the section where the temperature was 95 °C above the initial target temperature. Compared to the previous chip with the larger area heating pattern, it appears that excess energy is generated as the size of the heating pattern is reduced. To reduce this excess energy, we lowered the initial voltage (5 V) to 2 V or 2.5 V, which is equivalent to reducing the PWM control by 20% or 25%, respectively. In both cases, the DNA amplification was successful. However, the latter case is more efficient since it reduces the overall running time.

In this work, we conducted fluorescence detection using the micro-PCR chip. This included reducing the heater pattern area and applying the voltage appropriate for the optimal conditions of real-time PCR considering the reduced resistance of the heater pattern. The PCR performance shown was necessary for PWM control with different sizes of the heater pattern.

The organization of this paper is as follows. “Methods” section discusses the micro-PCR driving system and micro-PCR chip structure. This is followed by “Results and discussion” section presenting the results of real-time PCR at different voltages and, finally, “Conclusion” section.

## Methods

**Fig. 1:**
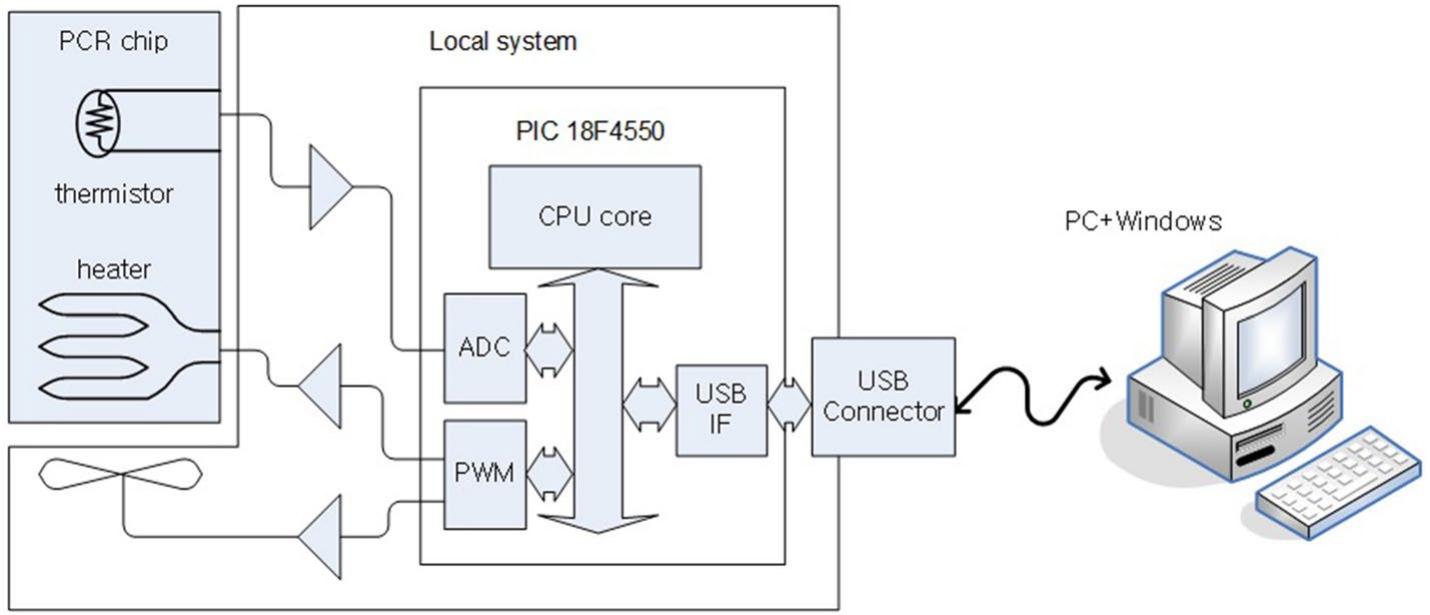
PCR chip and its control system

Figure 1 shows a block diagram of the proposed control system. The control system can be divided into a local system and the PCR chip. This paper focuses on the PCR chip component. The thermistor resistance of the PCR chip, originating from the voltage divider and ADC, is reported as a digital temperature value. A USB 2.0 port connects the local system to a PC. Every 50 ms, the temperature is recorded in the local system and the data values are sent to the host. At the host, the temperature values are read from the local system and the PWM value is calculated based on a proportional-integral-derivative (PID) controller mechanism. In other words, at the host, functions related not only to the temperature control but also to PCR protocol execution are processed through the graphical user inter-face (GUI) and the file input/output system [12, 13].

The control system of the PCR chip consists of biochemical and user interface functions. The biochemical function process consists of the protocol and the control of the chip temperature. The PCR protocol carries out actions that run repetitively at a set temperature and a certain time. Temperature sensing is used to control the chip’s temperature via the heater or the cooling fan. The basic biochemical function was designed and created to al-low direct management, editing, and control through the user interface. These two functions are integrated in the embedded system as one process. The system of local-host structures through the PC is shown in Fig. 1. This system structure was adopted because the embedded system can be easily controlled through the PC’s user interface. The temperature of the PCR chip was controlled using the temperature measurements and a periodic control procedure for the heater and fan. The PCR chip temperature was set to be increased or decreased by approximately 10 °C/s, and the processing period was set to control the temperature with an error range of less than 0.5 °C within 50 ms [14–18].

**Fig. 2:**
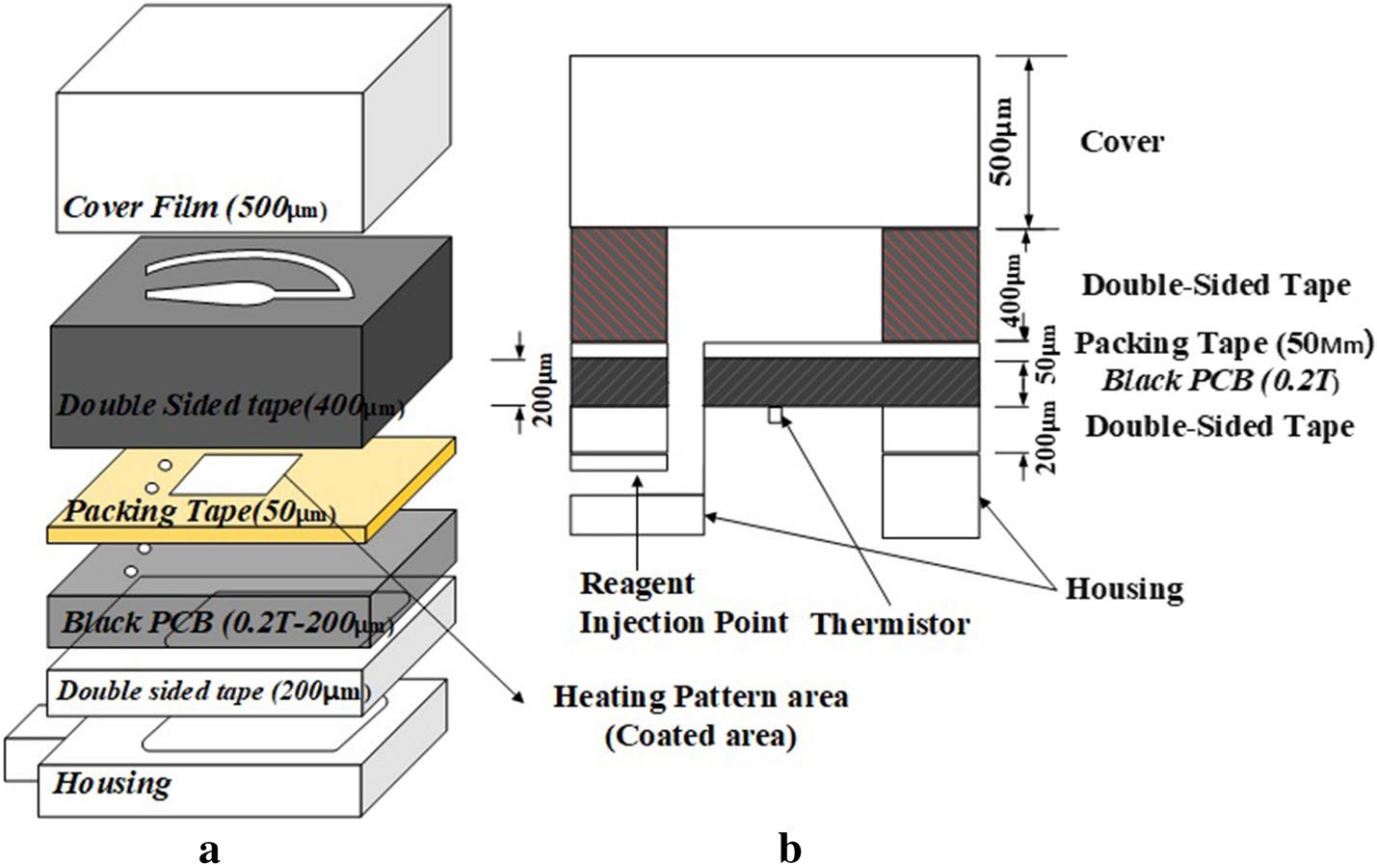
Structure of micro-PCR chip using black matte PCB. **a** Assembly diagram. **b** Cross section

The micro-PCR chip had a four-layer structure (black matte PCB, packing tape, double-sided tape, cover), with holes in the modified cover. However, the proposed micro-PCR chip here has been updated to a six-layer structure that contains housing and double-sided tape to connect the housing with the four-layered PCB chip, as shown in Fig. 2. A heating circuit is attached at the very bottom, with the PCB base and a thermal sensor for sensing temperature [19–22].

To prevent the fluorescence substance from sticking to the PCB, packing tape made of polypropylene material was attached to top of the PCB, and since the actual tube used in the laboratory was also made of polypropylene, the same material was also used for the chip to minimize sensitivity to the material [18, 19, 21]. To set the height of the microfluidic channel to 400 μm, 200 μm thick double-sided tape was attached in two layers. The chamber was fabricated by covering it with a 500 μm thick polypropylene material. With the micro-PCR chips previously reported, PCR was performed by making a microfluidic channel using regular green PCB, and the PCR performance was verified through electrophoresis.

With a real-time PCR system and a conventional chip structure, any light received by a fluorescent detector on green PCB is reflected and acts as noise. This negatively affects the measurement through the overall signal-to-noise ratio. To prevent reflection outside the chamber, here the cover was painted with a black marker pen as shown in Fig. 2, so that the chamber and other components could be easily identified.

Table 1 shows the tapes used in the experiment. In this work, packing tape and double-sided tape were used in the chip, with the double-sided tape serving as an important material for the channel in the micro-PCR chip (Product No. 5620BWN, Nitto Denko Co. Ltd., Japan). As reported in the past, it can withstand high temperatures of 95 °C and pressure when expanding. The adhesive was acrylic, and the carrier of the double-sided tape was composed of PET. In the tube-shaped structure shown in Fig. 2, injection of the reagent is facilitated through the inlet/outlet holes on both sides. The chamber in which the PCR was performed in the middle was fabricated by drilling a U-shaped hole.

**Table 1:** Packing and double-sided tapes used in the experiment

Heading level	Manufacturer	Tape number	Adhesive type
Packing tape	3M	#309 Mini Clear	Acrylic adhesive
Double-sided tape	Nitto Denko Co. Ltd. (Japan)	No. 5620BWN (double coated tape: 0.2 mm)	High adhesion acrylic

The cover at the top part of the chip was made with polypropylene and had a thickness of 500 μm. To reduce the reflection of blue LED light during detection, the top of the cover was colored with a black marker pen except for the reservoir. This enabled a more accurate observation of the reservoir compared to the case when the cover was not colored.

Lastly, the PCB and the upper structure were fixed with the housing, and this was used as a passageway for the reagent injection and the extraction of PCR products.

**Fig. 3:**
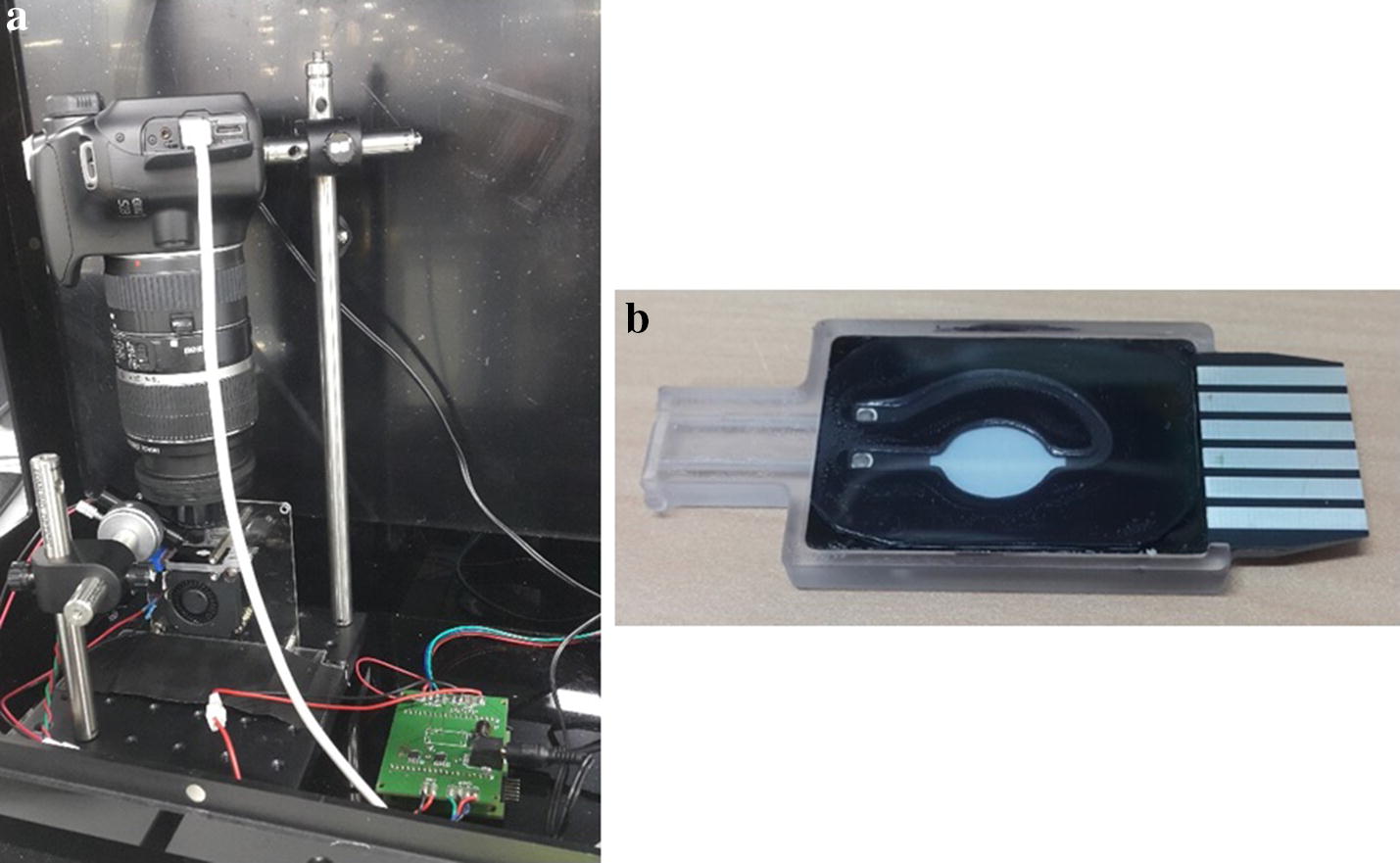
Fluorescence detection apparatus and micro-PCR chip. **a** Apparatus for real time PCR system. **b** Micro-PCR chip (black color microfluidic channel)

Figure 3a shows the fluorescence measurement apparatus used in the experiment. Images were captured with a DSLR camera (Canon 1100D). To detect FAM fluorescence, a blue LED (9600 mcd) was illuminated diagonally. Ideally, the light would be right above the observation area; however, it may cover the detection part, making it difficult to take measurements. Furthermore, despite the ease in setting up the lighting, there may be uneven light distribution. However, this would not affect the comparison analysis of overall fluorescent brightness, as the summations of the averaged mean values were used in the PCR image analysis. Figure 3b shows a micro-PCR chip fabricated with black PCB. As shown schematically in Fig. 2, the chip was fabricated into a structure that allows for easy observation of an increase or decrease of fluorescence brightness by printing a white-silk legend on the chamber to distinguish the fluorescence measurement.

In this experiment, the PCR process was performed according to the following protocol. To control the temperature in the proposed system, the PCR process, consisting of denaturation, annealing, and extension, was conducted as shown in Table 2. A total of 40 cycles were carried out for 3 min of pre-denaturing at 95 °C, 10 s at 95 °C, 30 s at 60 °C, and 30 s for 72 °C.

**Table 2:** PCR protocol used in the experiments

PCR process	Temperature (°C)	Time duration
1 cycle	95	3 min
40 cycles	95	10 s
	60	30 s
	72	30 s
1 cycle	95	10 s
	50	30 s

The reagent used for PCR comprised 1 ng/15.84 μL of DNA (*Chlamydia trachomatis*), 18 μL of Master Mix, 10 pM/0.72 μL of primer F, 10 pM/0.72 μL of primer R, and 10 pM/0.72 μL of Probe (total 36 μL).

The previously suggested heating pattern for the chip was large enough to heat the entire microfluidic channel. However, in this study, the large heating pattern (hereafter, large-sized pattern) increased the time to reach the target temperature, so the pattern was reduced to a smaller size (hereafter, small-sized pattern) that would only heat the reservoir area. However, when PCR was performed, even though only a short time was needed to reach the target temperature, DNA amplification did not occur. Since factors such as temperature, contamination, and material affect DNA amplification results during PCR, close monitoring was required to ascertain if the target temperature was accurately reached and if a constant temperature was sustained precisely for the duration indicated in the PCR protocol. A change in temperature was observed during the monitoring process, so PID control was used to control the temperature. During this process, the inside of the reservoir suddenly started boiling during the heating phase, at 25–95 °C (25 °C is not set in the PCR protocol, but it is set in the PID control process and may be regarded as the initial ambient temperature). This was thought to be caused by the small-sized heating pat-tern with very small resistance; consequently, the energy absorbed per unit area was larger than with the original. Therefore, the actual reagent temperature (internal temperature of the chip) would have been much higher than that indicated by the temperature sensor. This was important for reducing the oversupplied energy to the reagent. Consequently, the initial 5 V was reduced to both 2 V and 2.5 V using the power supply, and then PCR was performed. After reducing the power supply, the PID value was set to the initial value for the large-sized pattern for PCR. A proportional derivative gain (Kd) was not given in previous experiments, but since the heating pattern size was small and the temperature quite unstable while approaching the target temperature, a Kd value was given for these experiments.

**Fig. 4:**
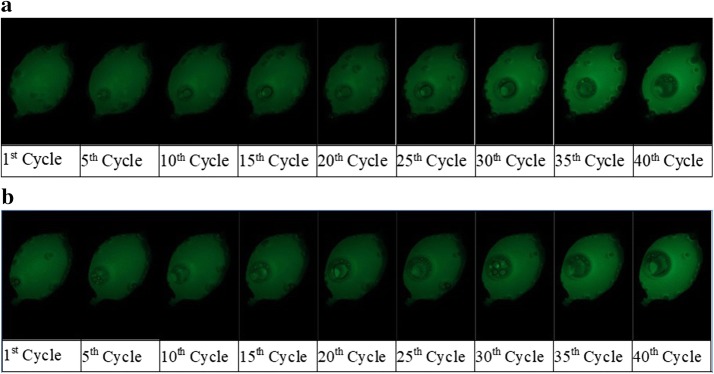
Changes of fluorescence brightness by PCR cycle. **a** 2 V power. **b** 2.5 V power

Figure 4 shows photographs of the changes in fluorescence brightness of the PCR cycle performed with the above PCR protocol. The experiment was conducted using the fluorescence photography setup shown in Fig. 3a, and the images were automatically captured with DSLR whenever a cycle proceeded at 72 °C (extension state of nucleic acid amplification); this is the middle amplification part of the PCR process, excluding the initial stage. As shown in the figure, it was confirmed that the fluorescence brightness increased as the PCR progressed. The total running time was 69 min 41 s (experiment conducted at 2 V) and 64 min 46 s (experiment conducted at 2.5 V). The total running time of the large-sized pat-tern was about 80 min, but this was decreased by 10–14 min after reducing the pattern size.

## Results and discussion

As shown in Fig. 4, the change in fluorescence brightness was insignificant at the early stages, but it started getting brighter halfway through the reaction and DNA amplification was also detected. To analyze the experimental results, the increase in fluorescence brightness was calculated in three different ways, using the sum of means, median, and Bhattacharyya distance, and these were plotted for a quantitative representation.

**Fig. 5:**
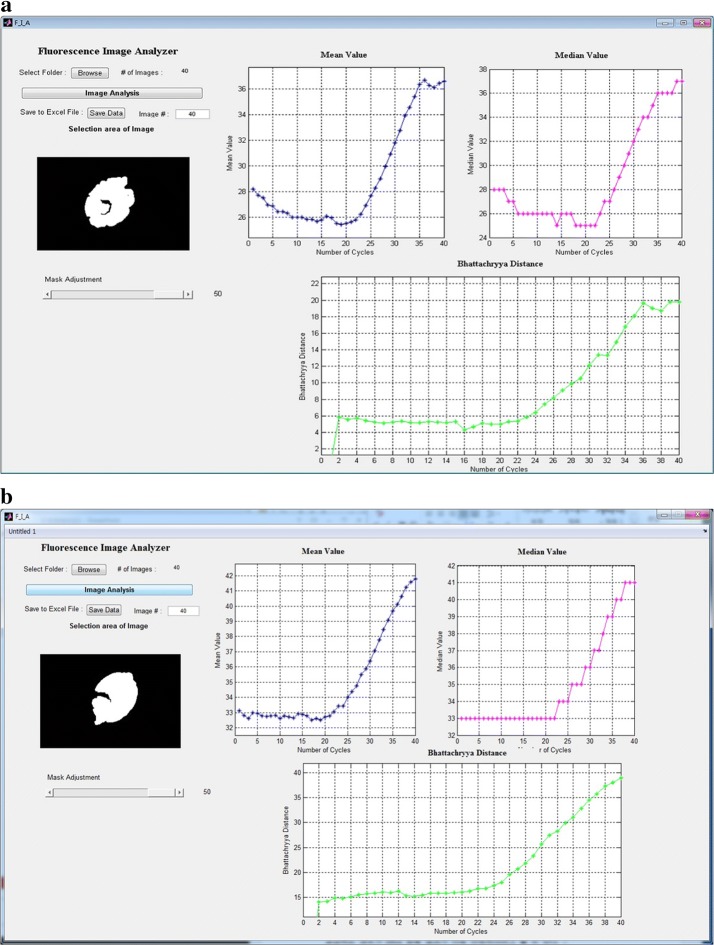
Real-time PCR result analysis by different voltage–power. **a** Fluorescence detection analysis results of voltage of 2 V (period 5, duty 1). **b** Fluorescence detection analysis results of voltage of 2.5 V (period 4, duty 1)

Figure 5 shows the analysis of real-time PCR results according to the different voltages. A Matlab program was used to analyze the fluorescence brightness from the 40 images captured with DSLR. As the pictures taken were in color, the images were changed to grayscale and the reservoir area was masked to calculate the sum of the mean, median, and Bhattacharyya distance. Each of these values was represented with a plot. This study used the sum of means as a brightness comparison measure for the fluorochrome. In the image processing field, this is the most general means for comparing brightness.

Here, it was thought that the sum of means was insufficient for a quantitative comparison of brightness. For a meaningful comparison of the brightness of the 40 images obtained during PCR, we compared and analyzed the images using the brightness histogram of images. The Bhattacharyya distance, which is a measure indicating image similarity, was used to compare before and after images. Plot trends similar to those for the sum of means were observed.

Based on the current reagent concentration levels, real-time PCR generally shows an insignificant change in fluorochrome brightness from the beginning of the PCR to cycle 19. However, many reports indicate an exponential increase beginning with cycle 20. Figure 5a, b respectively show the PCR results for the voltages of 2 V and 2.5 V. Both figures showed an exponential curve at cycle 20 with the sum of means, which is a typical result for real-time PCR with current concentration levels. Based on this, it can be concluded that excessive energy during the preheating and denaturation stage caused the reagent to have a higher temperature within the channel than that indicated by the NTC thermistor. This caused the DNA amplification to fail. Furthermore, based on the two experiments, heating and cooling were controlled using the PWM control method. The appropriate control method was determined by decreasing the voltage for the experiments. Considering various situations, DNA amplification in real-time PCR was successful when the initial volt-age was reduced from 5 to 2 V or 2.5 V. This effect was the same as when the voltage was reduced by turning it on only 20% or 25% of the time, while it is off the remaining time. The latter case had a shorter PWM control duty ratio, so it would have the more appropriate voltage level.

## Conclusions

Previously published studies indicated that a PCR test could be successful with micro-PCR chips made from double-sided tape. Furthermore, the green PCB was changed to a matte black PCB, and this minimized the noise. These enabled observations of a fluorochrome brightness change for each PCR cycle during real-time PCR. Reducing the time to attaining the target temperature will also reduce the overall PCR processing time. Therefore, the heating pattern size was decreased to more than one-quarter of the original size to reduce the heating pattern resistance and the time it takes to reach the target temperature. However, DNA amplification failed in this case, as the reagent was boiling in the chamber between 25 and 95 °C. Because the reduced resistance increased the energy per unit area, the actual reagent temperature in the chamber was higher than the temperature indicated by the NTC thermistor. This observation led to recognition of the importance of reducing the energy absorbed by the reagent. Heating and cooling were managed using PWM control, and the voltage was reduced and tested in order to find the appropriate PWM control method. The test had two conditions which reduced the maximum voltage to 2.5 V and 2 V. The voltage reduction has the same effect as reducing power by 25% and 20% using PWM control. Both cases had successful DNA amplification with real-time PCR. Typically, when a DNA concentration of 1 ng/μL is used, an exponential curve is observed from cycle 20. Here, an exponential increase was observed from cycle 20 at both lower voltages. However, the PWM control period was longer with 2 V; thus, using 2.5 V for PWM control should be more efficient. Based on these tests, using one-quarter of the voltage for PWM control (only 25% of PWM control is on) was selected for heating stages 25–95 °C, 60–72 °C, and 72–95 °C. DNA amplification was successfully achieved by reducing the duty ratio to 20% and 25% for PWM control. Afterward, the maximum value of PWM was subdivided into two ratios of 100% and 50%, and controlling experiments were conducted. Many factors determine the success of DNA amplification. Although it is important to achieve and preserve the tar-get temperature, it is also necessary to analyze for other factors, and more tests are needed to reduce the overall PCR processing time.

## Abbreviations

PCR: polymerase chain reaction
LOC: lab-on-a-chip
PCB: printed circuit board
PET: polyethylene terephthalate
ADC: analog-to-digital converter
FET: field-effect transistor
PWM: pulse width modulation
HPET: high-precision event timer
PID: proportional integral derivative
DNA: deoxyribonucleic acid
FAM: fluorescein amidite
PC: personal computer
NTC: negative temperature coefficient-thermic
DSLR: digital single-lens reflex
LED: light emitting diode
